# Digital design of a spatial-pow-STDP learning block with high accuracy utilizing pow CORDIC for large-scale image classifier spatiotemporal SNN

**DOI:** 10.1038/s41598-024-54043-7

**Published:** 2024-02-09

**Authors:** Mohammad Kazem Bahrami, Soheila Nazari

**Affiliations:** Faculty of Electrical Engineering, Shahid Beheshti University, Tehran, 1983969411 Iran

**Keywords:** Bio-Inspired SNN, AMPA and GABA neurotransmitters, LIF neurons, Image classification, Spatial-Pow-STDP, Unsupervised learning, Exp CORDIC, Ln CORDIC, Pow CORDIC, FPGA implementation, Machine learning, Computational neuroscience, Learning and memory, Neural circuits, Synaptic plasticity, Biomedical engineering, Electrical and electronic engineering

## Abstract

The paramount concern of highly accurate energy-efficient computing in machines with significant cognitive capabilities aims to enhance the accuracy and efficiency of bio-inspired Spiking Neural Networks (SNNs). This paper addresses this main objective by introducing a novel spatial power spike-timing-dependent plasticity (Spatial-Pow-STDP) learning rule as a digital block with high accuracy in a bio-inspired SNN model. Motivated by the demand for precise and accelerated computation that reduces high-cost resources in neural network applications, this paper presents a methodology based on COordinate Rotation DIgital Computer (CORDIC) definitions. The proposed designs of CORDIC algorithms for exponential (Exp CORDIC), natural logarithm (Ln CORDIC), and arbitrary power function (Pow CORDIC) are meticulously detailed and evaluated to ensure optimal acceleration and accuracy, which respectively show average errors near 10^–9^, 10^–6^, and 10^–5^ with 4, 4, and 6 iterations. The engineered architectures for the Exp, Ln, and Pow CORDIC implementations are illustrated and assessed, showcasing the efficiency achieved through high frequency, leading to the introduction of a Spatial-Pow-STDP learning block design based on Pow CORDIC that facilitates efficient and accurate hardware computation with 6.93 × 10^–3^ average error with 9 iterations. The proposed learning mechanism integrates this structure into a large-scale spatiotemporal SNN consisting of three layers with reduced hyper-parameters, enabling unsupervised training in an event-based paradigm using excitatory and inhibitory synapses. As a result, the application of the developed methodology and equations in the computational SNN model for image classification reveals superior accuracy and convergence speed compared to existing spiking networks by achieving up to 97.5%, 97.6%, 93.4%, and 93% accuracy, respectively, when trained on the MNIST, EMNIST digits, EMNIST letters, and CIFAR10 datasets with 6, 2, 2, and 6 training epochs.

## Introduction

Realizing energy-efficient computing systems has become a paramount concern for machines with significant cognitive capabilities. Mammalian cortexes exhibit remarkable efficiency, consuming a mere 10 to 20 watts during high-level cognitive processes^1^, a stark contrast to contemporary computer systems engaged in analogous functions. This pronounced disparity in energy utilization has propelled the quest for energy-efficient computing solutions. In recent years, notable endeavors have focused on implementing Spiking Neural Networks (SNNs) on neuromorphic chips^2^, where energy consumption has been reduced to the picojoule scale in the transmission of each spike. This achievement has rendered neuromorphic hardware conducive for on-chip learning^3^ mechanisms and is elevating SNNs to paramount importance in machine learning and artificial intelligence applications^4^.

Parallel to this, deep learning techniques have gained considerable prominence in the scientific community, primarily through the adoption of deep neural networks (DNNs) for supervised and reinforcement learning tasks^5^. Among DNNs, convolutional neural networks (CNNs) have excelled in automatically extracting features from input data. However, SNNs operating on an event-driven, asynchronous paradigm demonstrate significantly lower power consumption than their DNN counterparts^6,7^. Herein, a novel spiking pattern recognition platform contains a biologically possible SNN and a bio-inspired learning approach based on extended versions of Spike Time Dependent Plasticity (STDP). This platform exhibits superior accuracy and learning speed compared to previous SNN-based pattern recognition systems and attains accuracy levels akin to deep pattern recognition networks, with additional advantages such as compatibility with neuromorphic chips, unsupervised learning, and higher convergence speed. The remarkable ability of the designed spiking platform for pattern recognition can be seen in the novel variant of STDP called Spatial Power Spike-Timing-Dependent Plasticity (Spatial-Pow-STDP).

One typical dataset used to evaluate network performance is the MNIST dataset^8^, which has been successfully classified by deep conventional neural networks. In addition to MNIST, the proposed network's performance on an extended version of MNIST called EMNIST^9^, which includes handwritten digits and letters, was evaluated. Also, the dataset CIFAR10^10^, which is very challenging to classify by unsupervised networks, has been investigated to verify the proposed learning performance. Notably, achieving high accuracy on EMNIST and CIFAR10 using spiking pattern recognition networks remains a formidable challenge^11,12^, marking this work as state-of-the-art. While mapping deep networks to spiking networks and enhancing classification accuracy with supervised spike-based back-propagation have been explored in recent studies^13^, the proposed platform compared to previous spiking networks indicates several advantages, including higher classification accuracy, faster convergence speed, an unsupervised training method, fewer hyper-parameters, and network layers.

FPGA-based implementations of spiking neural networks have been explored for a variety of applications including pattern recognition^14,15^, context-dependent learning^14^, and auditory processing^16^. For pattern recognition tasks, spiking neural networks using leaky integrate-and-fire neuron models and spike timing dependent plasticity learning rules have been implemented on FPGAs to achieve high speeds such as 189 MHz^17^ and 412 MHz maximum frequency for a single neuron model^18^. Other works have focused on implementing spiking neural networks with reinforcement learning capabilities for context-dependent learning tasks, using modified leaky integrate-and-fire models to enable faster convergence and lower power consumption compared to previous FPGA implementations^18^. In the auditory domain, FPGA implementations of spiking neural networks have aimed to mimic auditory pathways in the brain, using bio-inspired hierarchical network architectures and biological neuron models to achieve robustness against noise^19^. Overall, FPGAs provide a flexible platform for spiking neural network implementation to leverage the benefits of event-driven, parallel, and low power processing for time-critical and power-constrained applications^19–21^. In works focused on the hardware implementation of the pattern recognition networks^14,15^, new network topologies and learning models were not presented. In such works, hardware implementation was done for networks whose structure and learning model had already been presented in other works. In contrast, our work focused on two key principles: (1) Providing a hardware learning module based on CORDIC with high accuracy. (2) Proposing a spiking network with unsupervised learning modeled on AMPA and GABA synapses to identify MNIST, EMNIST, and CIFAR10 patterns with higher accuracy and faster convergence than previous spiking and deep neural networks.

Moreover, this study proposes a digital design and evaluation method for the Spatial-Pow-STDP learning module based on COordinate Rotation DIgital Computer (CORDIC) to address critical issues related to hardware efficiency and learning accuracy. This comprehensive approach spans from theoretical CORDIC-level error analysis to application-level learning performance on MNIST, EMNIST, and CIFAR10 datasets. The goal is to identify the optimal CORDIC type among various algorithms and the lowest bit-width precision to maximize overall SNN hardware efficiency while minimizing performance loss and hardware overhead^22^. Within this context, a digital module of Spatial-Pow-STDP based on a selected 16-bit Pow CORDIC is presented, and Field-Programmable Gate Array (FPGA) implementation results confirm its superiority over conventional and state-of-the-art CORDIC methods in terms of hardware efficiency.

In neural network computation, logarithms and exponentials play a pivotal role, particularly in SNNs. Two primary approaches exist for evaluating logarithms and exponentials: approximation^23^ and iterative methods^24^. While these methods have been considered in many research studies, this paper introduces a promising solution to enhance hardware efficiency by improving the computation of logarithms, exponentials, and power functions using a novel CORDIC algorithm. Specifically, this paper introduces a novel natural logarithm and exponential calculation using the CORDIC algorithm, renowned for its high accuracy and speed. Leveraging these enhanced natural logarithms and exponential CORDIC techniques, a novel CORDIC variant, called Pow CORDIC, is introduced for calculating power functions. The Pow CORDIC block can calculate power functions with arbitrary power terms, a critical requirement in the second and third generation of neural networks.

We evaluate these CORDIC-based techniques and implement them on an FPGA using the VHDL language, examining their resource consumption. The Pow CORDIC is used to implement the Spatial-Pow-STDP learning module efficiently, which is the proposed training module in the presented spiking pattern recognition network. The classification accuracy of the software (Spatial-Pow-STDP) and hardware (CORDIC-based Spatial-Pow-STDP) learning blocks are assessed by the MNIST, EMNIST, and CIFAR10 datasets, revealing the potential benefits of this innovative methodology in advancing neural network hardware efficiency and learning performance.

In summary, this paper signifies a pivotal convergence of advancements in energy-efficient computing and bio-inspired neural networks. It introduces groundbreaking techniques culminating in a novel and highly efficient Spatial-Pow-STDP learning rule for bio-inspired SNNs. Moreover, this research pioneers the development of more accurate and faster natural logarithm CORDIC and exponential CORDIC algorithms, facilitating the precise calculation of power functions with arbitrary power terms using Pow CORDIC. These innovations synergize to empower the implementation of Spatial-Pow-STDP, propelling the efficiency and accuracy of neural network modeling to unprecedented levels. This multifaceted approach not only advances energy-efficient neuromorphic hardware but also opens new horizons for the precision and efficiency of neural network computations.

## Computational SNN model

In this research, a cutting-edge SNN model is adopted, built upon Leaky Integrate-and-Fire (LIF) neurons^25^, including both pyramidal and interneurons. Additionally, it incorporates an unsupervised STDP variant learning rule^26^. The choice of this SNN architecture and learning rule is based on their proven effectiveness in prior studies^27,28^. This particular SNN framework, coupled with the selected learning rule, has demonstrated its ability to achieve superior accuracy when evaluated against well-established dataset benchmarks. Furthermore, it exhibits a notable advantage in terms of the speed at which it converges during the learning process, making it a robust choice for this study.

### Computational model of excitatory and inhibitory neurons and synapses

The representation of neurons (including both pyramidal neurons and interneurons) within the innovative SNN utilizes LIF neurons. In this context, the membrane potential, denoted as ${v}_{k}$, is characterized as follows:

1 $$\begin{array}{c}{\tau }_{m}\frac{d{v}_{k}(t)}{dt}=-{v}_{k}\left(t\right)+[{V}_{Ak}\left(t\right)-{V}_{Gk}\left(t\right)]\end{array}$$

The representation of the excitatory post-synaptic potential (AMPA potential) is described as ${V}_{Ak}$ in Eq. (2), which is the product of membrane resistance and AMPA synaptic current. In Eq. (3), the combined effect of excitation from pyramid neurons linked to neuron $k$ and the stimulating input to neuron $k$ is computed as an auxiliary variable called ${x}_{Ak}$. This auxiliary variable ${x}_{Ak}$ is then utilized as the excitatory input for generating the AMPA potential ${V}_{Ak}$.

2 $$\begin{array}{c}{\tau }_{dA}\frac{{dV}_{Ak}}{dt}=-{V}_{Ak}+{x}_{Ak}\end{array}$$

3 $$\begin{array}{c}{\tau }_{rA}\frac{{dx}_{Ak}}{dt}={-x}_{Ak}+{\tau }_{m}\left({J}_{k-pyr}\sum_{pyr}\delta (t-{t}_{k-pyr}-{\tau }_{L})+{J}_{k-ext}\sum_{ext}\delta (t-{t}_{k-ext}-{\tau }_{L})\right)\end{array}$$

Likewise, the representation of the inhibitory post-synaptic potential (GABA potential) is denoted as ${V}_{Gk}$ in Eq. (4), which is the product of membrane resistance and GABA synaptic current. The cumulative impact of inhibition originating from the interneurons connected to neuron $k$ is computed in Eq. (5) as an auxiliary variable termed ${x}_{Gk}$. This auxiliary variable ${x}_{Gk}$ is then employed as an input in Eq. (4) to generate the GABA potential ${V}_{Gk}$.

4 $$\begin{array}{c}{\tau }_{dG}\frac{{dV}_{Gk}}{dt}=-{V}_{Gk}+{x}_{Gk}\end{array}$$

5 $$\begin{array}{c}{\tau }_{rG}\frac{{dx}_{Gk}}{dt}={-x}_{Gk}+{\tau }_{m}\left({J}_{k-int}\sum_{int}\delta (t-{t}_{k-int}-{\tau }_{L})\right)\end{array}$$

The parameters ${\tau }_{m}$ (20 ms for excitatory neurons and 10 ms for inhibitory neurons), ${v}_{thr}$ (18 mV), ${v}_{res}$ (0 mV), ${\tau }_{rp}$ (2 ms for excitatory neurons and 1 ms for inhibitory neurons), and ${\tau }_{L}$ (1 ms) are defined as follows: ${\tau }_{m}$ represents the membrane time constant, ${v}_{thr}$ stands for the threshold at which neurons fire, ${v}_{res}$ is the resting potential, ${\tau }_{rp}$ is the refractory time, and ${\tau }_{L}$ denotes the latency of post-synaptic potentials. Additionally, terms like ${t}_{k-pyr,int,ext}$, ${\tau }_{dA}$ (or ${\tau }_{dG}$), and ${\tau }_{rA}$ (or ${\tau }_{rG}$) are used to describe the timing of received spikes from pyramidal neurons, interneurons, and external inputs to neuron $k$, as well as the decay and rise times of excitatory (or inhibitory) AMPA (or GABA) synaptic potentials. The learning within the network heavily relies on excitatory and inhibitory synaptic weights, which are represented by ${J}_{k-pyr}$ (for excitatory synapses from pyramidal neurons to neuron $k$), ${J}_{k-int}$ (for inhibitory synapses from interneurons to neuron $k$), and ${J}_{k-ext}$ (for excitatory synapses from external inputs to neuron $k$). However, the excitatory synapse ${J}_{k-ext}$ is static and does not participate in learning. The values of parameters are adopted from Ref.^28^.

In this paper, due to the simplification of network dynamics, dendritic connections are not considered in the computational model of pyramidal neurons. If dendritic connections were used in the pyramidal neuron model, the amount of computation would be greatly increased and complexity would arise in the training process. Instead of dendritic connections, excitatory and inhibitory synapses of AMPA and GABA neurotransmitters have been used in modeling neuronal interactions.

**Figure 1:**
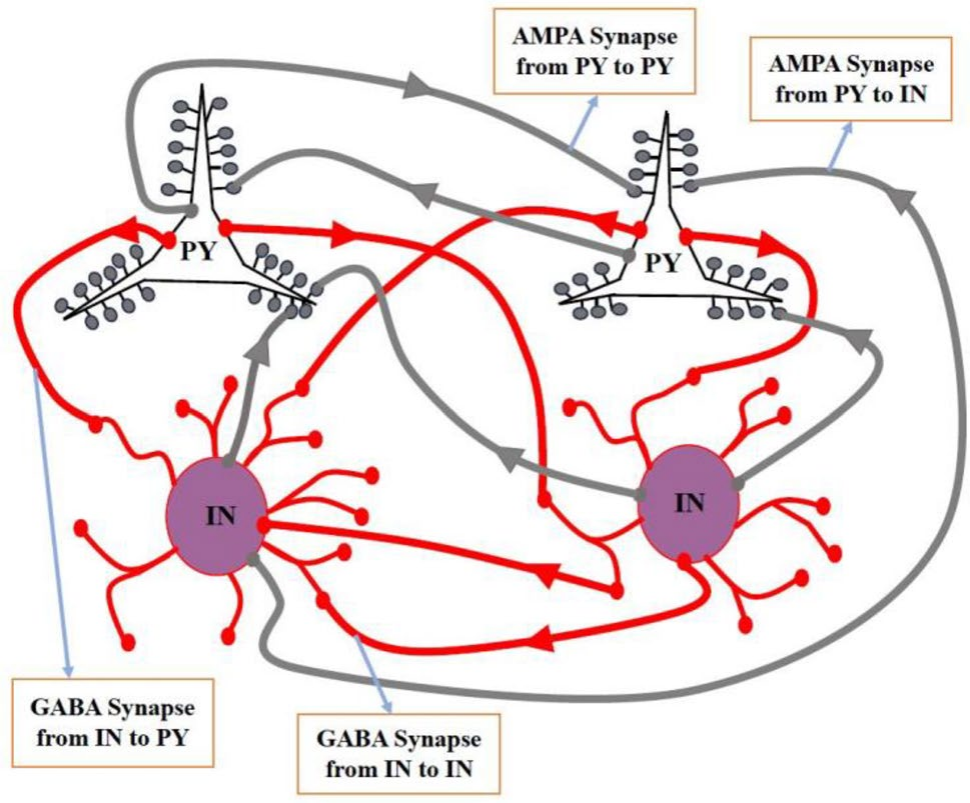
Pyramidal neurons (PYs) transmit stimulating signals to connected neurons through excitatory neurotransmitters, while interneurons (INs) relay inhibitory signals to connected neurons via inhibitory neurotransmitters.

In general, pyramidal neurons and interneurons are characterized using the LIF neuron model as outlined in Eq. (1). The modeling of excitatory AMPA synaptic potentials is explained by Eq. (2) and Eq. (3), while inhibitory GABA potentials are described by Eq. (4) and Eq. (5). Pyramidal neurons, through excitatory neurotransmitters represented by Eq. (3), stimulate their post-synaptic neurons via AMPA synapses defined in Eq. (2). Similarly, interneurons, employing inhibitory neurotransmitters as per Eq. (5), inhibit their post-synaptic neurons through GABA synapses as per Eq. (4). The neural interactions encompass interactions between interneurons, pyramidal neurons, and interneurons to pyramidal neurons, as depicted in Fig. 1.

### Architecture of spiking image classification networks

The spiking image classification network comprises a retinal model as the input layer, followed by a bio-inspired spiking neural network as the middle layer, with pyramidal neurons serving as the classifying neurons.

#### Input layer

The MNIST dataset consists of 28 × 28 images that show digits from 0 to 9 (with a total of 60,000 training images and 10,000 test images). Also, the EMNIST dataset encompasses images sized at 128 × 128 pixels featuring digits from 0 to 9 (with 240,000 training patterns and 40,000 test patterns) and letters spanning A to Z (comprising 88,800 training patterns and 14,800 test patterns). Meanwhile, the CIFAR10 dataset consists of 10 classes of natural images, each measuring 32 × 32 pixels (with 50,000 training patterns and 10,000 test patterns).

In contrast to prior studies^29^, where the size of the input layer scaled with the dimensions of the input image, in this case, input patterns are transformed to spike trains based on the bio-inspired visual pathway. As a result, the patterns from the MNIST, EMNIST, and CIFAR10 datasets, respectively are converted into 7 × 7, 8 × 8, and 8 × 8 spike trains, enabling a significant reduction in the size of the input layers for the MNIST, EMNIST, and CIFAR10 classification networks.

**Figure 2:**
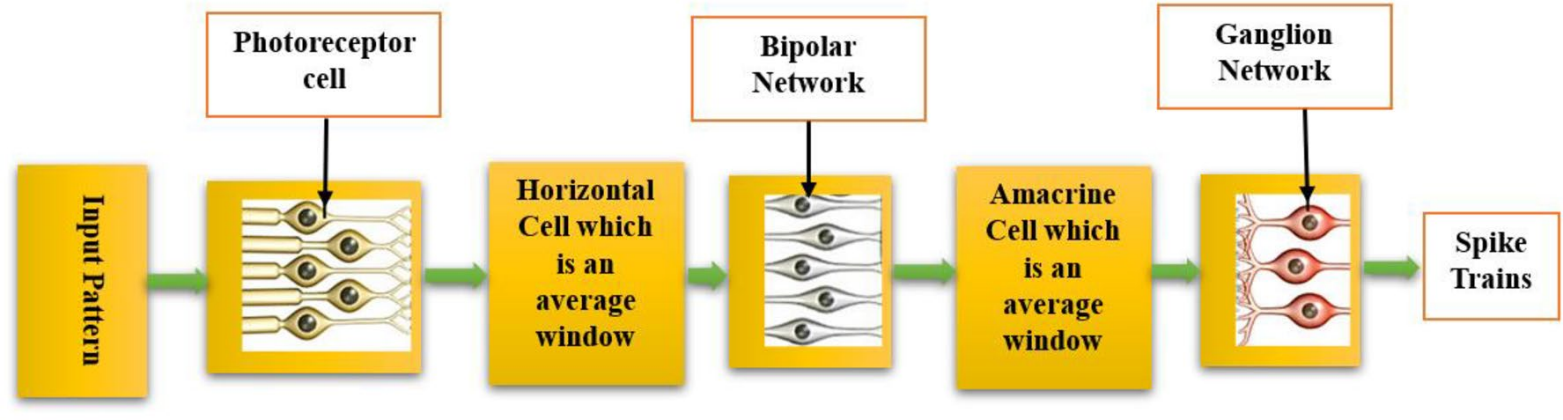
Conversion of input patterns into the spike trains is employed to minimize the input layer dimensions within the pattern classification networks.

In the input layer, MNIST, EMNIST, and CIFAR10 images are initially processed by photoreceptors. Subsequently, these images are routed through horizontal cells, where they undergo averaging with a 2 × 2 window and a stride of 2, resulting in electrical signals of sizes 14 × 14, 64 × 64, and 16 × 16, respectively. These electrical signals then pass through bipolar cells^30^, where they are subjected to further averaging using an 8 × 8 window with an 8-pixel stride for EMNIST and a 2 × 2 window with a stride of 2 for MNIST and CIFAR10, leading to electrical signals sized 7 × 7, 8 × 8, and 8 × 8 for MNIST, EMNIST, and CIFAR10, respectively. Finally, ganglion cells^31^ transform these electrical signals from amacrine cells into 7 × 7, 8 × 8, and 8 × 8 spike trains. This process is generally shown in Fig. 2. These spike trains are stimuli for the pyramidal and interneurons in the middle layer through excitatory synapses. The dynamic models of the ON/OFF bipolar and ganglion networks are interconnected using the model outlined in a previous paper^32^ to construct the retinal model.

#### Middle layer

The middle layer of the EMNIST and CIFAR10 classification networks consists of N = 5000 neurons. Among these, 80% are pyramidal neurons (PY), which are excitatory, and the remaining 20% are interneurons (IN), which are inhibitory. Consequently, in the MNIST recognition network, there are 4000 PY and 1000 IN neurons, and the same distribution applies to the EMNIST recognition network. The complexity of this spiking network is determined by the intricate dynamics of synapses and the extent of neural communication. In this setup, all network neurons are based on the LIF neuron model, and the neural interactions are simulated using dynamic models of excitatory and inhibitory neurotransmitters such as AMPA and GABA, as shown in Fig. 1.

In addition to considering the dynamics of excitatory and inhibitory synapses within the spiking network, the number of synapses present in the network plays a significant role in shaping its learning capabilities. Implementing pattern recognition networks on neuromorphic hardware platforms presents challenges such as high power consumption, complex implementation, and speed limitations, especially when utilizing fully connected networks. Fortunately, research has demonstrated that reducing the density of connections in fully connected networks by up to 90% and transitioning to a sparsely connected network architecture can enhance performance accuracy. Additionally, experimental evidence suggests that the connection probability between two neurons in the nervous system is approximately 0.2^33^.

**Figure 3:**
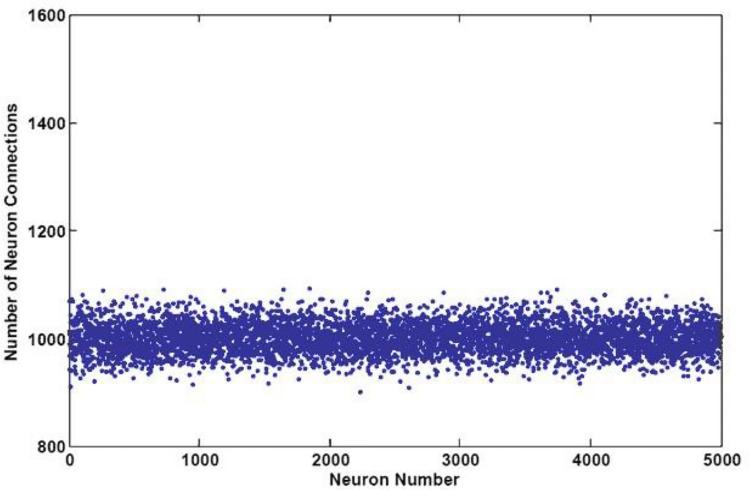
The count of connections (comprising both excitatory AMPA and inhibitory GABA synapses) per neuron within the EMNIST classification network.

Considering a connection probability of 0.2 within the middle layer, each neuron establishes connections with roughly 1000 other neurons. Therefore, in the middle layer of the MNIST, EMNIST, and CIFAR10 classification networks, we have integrated an excess of 5 million excitatory and inhibitory synapses, precisely 5,123,640 synapses for MNIST, 5,100,762 for EMNIST, and 5,074,150 for CIFAR10. Figure 3 illustrates the synapse counts per neuron in the middle layer of the EMNIST classification network.

Considering that the transmission of information between neurons is influenced not only by the timing of pre- and post-synaptic spikes but also by the spatial distance between neurons, we have organized neurons within a rectangular area measuring 100 by 50 neurons^27^. In this context, the intensity of interactions between neurons is represented as diminishing exponentially with greater distances, as denoted by the term ${e}^{\frac{-r}{D}}$ incorporated into Eqs. (3) and (5) are the dynamic equations governing excitatory and inhibitory synapses. This modification results in Eq. (6) and (7) are presented as below:

6 $$\begin{array}{c}{\tau }_{rA}\frac{{dx}_{Ak}}{dt}={-x}_{Ak}+{\tau }_{m}\left({e}^{\frac{-r}{D}}{J}_{k-pyr}\sum_{pyr}\delta (t-{t}_{k-pyr}-{\tau }_{L})+{J}_{k-ext}\sum_{ext}\delta (t-{t}_{k-ext}-{\tau }_{L})\right)\end{array}$$

7 $$\begin{array}{c}{\tau }_{rG}\frac{{dx}_{Gk}}{dt}={-x}_{Gk}+{{e}^{\frac{-r}{D}}\tau }_{m}\left({J}_{k-int}\sum_{int}\delta (t-{t}_{k-int}-{\tau }_{L})\right)\end{array}$$

In Eq. (6), $r$ represents the distance between the pre-synaptic pyramidal neurons and neuron $k$, and in Eq. (7), $r$ signifies the distance between the pre-synaptic interneuron and neuron $k$. Here, $D$ denotes the constant for scaling distances. The term ${e}^{\frac{-r}{D}}$ specifically influences the interactions between neurons by modulating the strength of both excitatory and inhibitory synapses. Notably, this term does not impact input synapses because the input spike train stimulates pyramidal neurons and interneurons regardless of the distance between neurons and input nodes. Hence, in Eq. (6), ${e}^{\frac{-r}{D}}$ is enclosed within parentheses to denote its specific applicability.

#### Classifier layer

**Figure 4:**
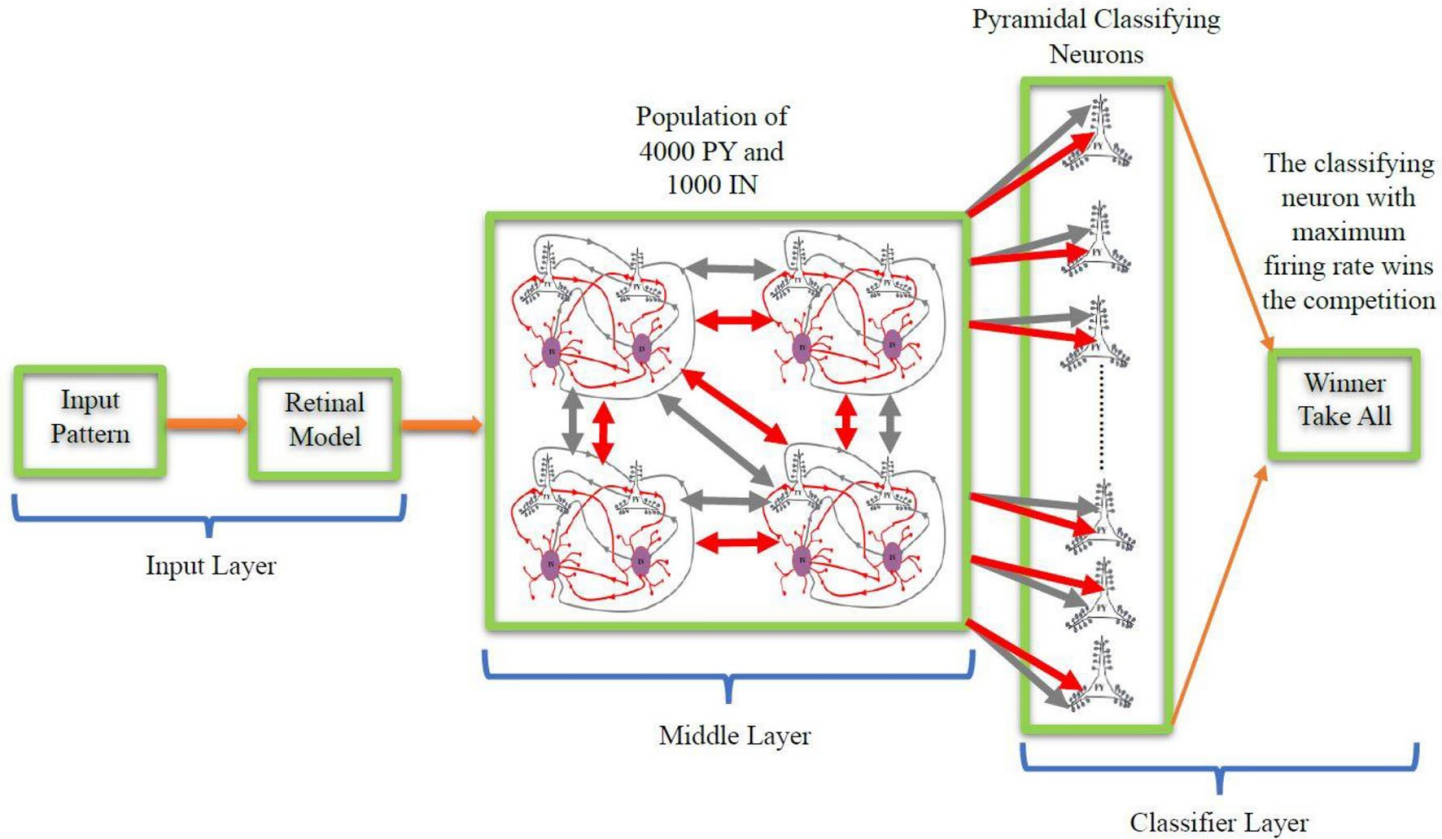
The general architecture of the image classification networks consists of an initial input layer represented by a retinal model, followed by a bio-inspired SNN in the middle layer, and subsequently, with a group of classifying neurons forming the classifier layer.

In alignment with the 36 classes in EMNIT and the 10 classes in MNIST and CIFAR10, an equal number of LIF pyramidal neurons are positioned in the classifier layer of their respective pattern classification networks. These classifying neurons are linked to all pyramidal neurons in the middle layer through excitatory connections and to the interneurons in the middle layer via inhibitory connections. Each neuron in the classifier layer corresponds to a specific class within the training dataset, thereby assigning the responsibility of classifying test data to the neurons of this layer after the training phase. The classifying neuron exhibiting the highest firing rate signifies the winning class during the winner-takes-all process. The general network structure for the MNIST, EMNIST, and CIFAR10 classification networks is illustrated in Fig. 4.

Utilizing the retinal model within the input layer of pattern classification networks offers a novel approach to reduce dimensionality while safeguarding the integrity of input data. This feature can be valuable in various other machine learning applications. Maintaining the image information during the conversion to spike trains within the input layer of pattern classification networks is a pivotal aspect of the learning process, as each pattern is uniquely represented by its corresponding spike train.

### Learning mechanism

Recent research has emphasized the importance of developing learning mechanisms tailored to SNNs, drawing inspiration from the neural interactions in the nervous system. Hebbian learning and STDP mechanisms^34^ have been prominent due to their biological relevance and integration into SNNs. While supervised methods like spike-based back-propagation^13^ and transformed spiking deep networks^35^ exist, unsupervised STDP learning is crucial due to its alignment with biological evidence. Due to the high cognitive ability of the nervous system, to enhance the capability learning of machines, aligning machine calculations with neural spike calculations is essential^36^. Recent efforts have led to the development of SNNs, mirroring the brain's functional structure^4^. These networks rely on temporal information coding and spatially distributed neuronal populations for learning^4^. Hence, this paper presents a novel adaptation of the spike-timing-dependent plasticity mechanism derived from a variant of the STDP rule characterized by a weight-dependent update equation based on a power-law function^27^. This modified mechanism is referred to as "spatial power spike-timing-dependent plasticity (Spatial-Pow-STDP)," and it influences the transmission of AMPA and GABA neurotransmitters as part of the learning process^37^. This approach aligns with biological evidence and updates synaptic weights based on the timing of spike activity of pre-and post-synaptic neurons and their spatial distance until patterns are stored in network memory.

Within the framework of Spatial-Pow-STDP learning, as illustrated in Eq. (8), there is an optimization aimed at enhancing simulation speed by computing weight dynamics through synaptic traces. In addition to monitoring synaptic weight, each synapse also maintains another parameter known as the pre-synaptic trace, denoted as ${x}_{pre}$, which reflects the recent history of pre-synaptic spikes. As detailed in Eq. (9), whenever a pre-synaptic spike reaches the synapse, this trace increases by 1; otherwise, it undergoes exponential decay. Subsequently, when a post-synaptic spike occurs at the synapse, the weight change ($\Delta w$) is determined based on the pre-synaptic trace^29,38^.

8 $$\begin{array}{c}\Delta w={e}^{\frac{{\text{r}}}{{{\text{D}}}_{{\text{s}}}}} \eta \left({x}_{pre}-{x}_{tar}\right){\left({w}_{max}-w\right)}^{\mu }\end{array}$$

9 $$\begin{array}{c}{\tau }_{{x}_{pre}}\frac{d{x}_{pre}}{dt}= -{x}_{pre}+\sum_{pre}\delta (t-{t}_{pre})\end{array}$$

In Eqs. (8) and (9), several key parameters are defined. These include $\eta$ as the learning rate, $w$ representing synaptic weight within the range of 0 to 1, ${w}_{max}$ as the maximum weight, ${\tau }_{{x}_{pre}}$ serving as the time constant for ${x}_{pre}$, and $\mu$, a positive value less than 1, determining the influence of the previous weight on updates. Additionally, ${x}_{tar}$ signifies the target value for the pre-synaptic trace at the moment of a post-synaptic spike.

Furthermore, the variable $r$ denotes the distance between two pre- and post-synaptic neurons that have fired, with ${D}_{s}$ serving as a constant parameter representing the scale of this distance. Essentially, the terms involving ${e}^\frac{r}{Ds}$ capture the spatial characteristics within the learning process. This spatial learning component expedites the network's learning convergence for two primary reasons: Firstly, when two neurons are distant from each other and have short spike intervals, they exhibit a greater increase in synaptic weight compared to standard STDP. Secondly, when two neurons are nearby and have short spike intervals, their synaptic weight increase is less than what is observed with STDP.

The measurement of pre-synaptic spiking activity over a time window denoted as ${x}_{pre}$, effectively represents the dynamic of synaptic strength between two neurons through STDP. As depicted in Eq. (8), when a post-synaptic neuron fires and ${x}_{pre}$ is larger, it signifies a smaller time difference between the spiking of the two neurons, resulting in a more substantial contribution from the pre-synaptic neuron. Consequently, $\Delta w$ increases significantly, strengthening the connection between the neurons, a phenomenon known as long-term potentiation (LTP). Conversely, when the pre-synaptic neuron's contribution to the post-synaptic neuron's firing is minimal, and the spiking time difference is substantial, $\Delta w$ can become negative to weaken the synaptic connection. This adjustment is achieved with the assistance of the target trace ${x}_{tar}$, reflecting long-term depression (LTD).

Understanding cellular processes and the dynamic principles governing interactions within the nervous system is challenging because of the vast diversity of biological cells and the intricate nature of neural synapses that transmit information. To gain insight into the mechanisms underlying synaptic weight changes and synaptic plasticity in the learning process, it is essential to investigate and uncover the structure and functional behavior of ion channels and neurotransmitters. A significant discovery in this context is the impact of neurotransmitters like AMPA (excitatory) and GABA (inhibitory) on regulating synaptic weight changes, ultimately influencing synaptic plasticity^37^.

The transmission of AMPA neurotransmitters within the synaptic space is a pivotal factor in information storage and learning within the nervous system. Manipulating AMPA neurotransmitter levels by increasing or decreasing them has been shown to induce STDP during learning. Similarly, the modulation of GABA neurotransmitters also significantly influences synaptic weight adjustments, consequently impacting STDP and thereby influencing learning processes. Consequently, the Spatial-Pow-STDP learning approach is defined by employing equations that describe the dynamics of AMPA and GABA potentials, which simulate the learning processes through regulating AMPA and GABA neurotransmitter transmission levels.

As a result, the learning process is guided by four distinct rules.

1. When connecting a pyramidal neuron to another pyramidal neuron, an initial weight is set randomly. If the post-synaptic neuron fires after the pre-synaptic neuron, the AMPA synaptic weight between them increases according to ${e}^{\frac{r}{{D}_{s}}} {\eta }_{PY}\left({x}_{pre}-{x}_{tar}\right){\left({w}_{max}-w\right)}^{\mu }$ (Eq. (10)).
2. The initial weight is determined randomly when establishing a connection from an interneuron to a pyramidal neuron. If the post-synaptic neuron fires after the pre-synaptic neuron, the GABA synaptic weight between them decreases according to ${e}^{\frac{r}{{D}_{s}}} {\eta }_{PY}\left({x}_{pre}-{x}_{tar}\right){\left({w}_{max}-w\right)}^{\mu }$ (Eq. (11)).
3. The initial weight is assigned randomly when forming a connection from a pyramidal neuron to an interneuron. If the post-synaptic neuron fires after the pre-synaptic neuron, the AMPA synaptic weight between them increases based on ${e}^{\frac{r}{{D}_{s}}} {\eta }_{IN}\left({x}_{pre}-{x}_{tar}\right){\left({w}_{max}-w\right)}^{\mu }$ (Eq. (12)).
4. When connecting an interneuron to another interneuron, the initial weight is determined randomly. If the post-synaptic neuron fires after the pre-synaptic neuron, the GABA synaptic weight between them decreases based on ${e}^{\frac{r}{{D}_{s}}} {\eta }_{IN}\left({x}_{pre}-{x}_{tar}\right){\left({w}_{max}-w\right)}^{\mu }$ (Eq. (13)).

It is important to mention that random synaptic weights are based on Gaussian distribution with zero mean and 0.2 standard deviation, that excitatory and inhibitory synapses have positive weight, but the effect of AMPA excitatory synapses on post-synaptic neuron stimulation is positive and the effect of inhibitory synapses is negative. The subsequent equations (Eqs. (10) and (11) for pyramidal neurons and Eqs. (12) and (13) for interneurons) depict the mathematical expressions that govern the learning process in pattern classification networks:

10 $$\begin{array}{c}{\tau }_{rA}\frac{{dx}_{Ak}}{dt}={-x}_{Ak}+{\tau }_{m}\left(\begin{array}{c}{e}^{\frac{-r}{D}}\left({J}_{k-pyr}+{e}^{\frac{r}{{D}_{s}}} {\eta }_{PY}\left({x}_{pre}-{x}_{tar}\right){\left({w}_{max}-w\right)}^{\mu }\right)\sum_{pyr}\delta (t-{t}_{k-pyr}-{\tau }_{L})\\ +{J}_{k-ext}\sum_{ext}\delta (t-{t}_{k-ext}-{\tau }_{L})\end{array}\right)\end{array}$$

11 $$\begin{array}{c}{\tau }_{rG}\frac{{dx}_{Gk}}{dt}={-x}_{Gk}+{\tau }_{m}\left({e}^{\frac{-r}{D}}\left({J}_{k-int}-{{\text{e}}}^{\frac{{\text{r}}}{{{\text{D}}}_{{\text{s}}}}} {\eta }_{PY}\left({x}_{pre}-{x}_{tar}\right){\left({w}_{max}-w\right)}^{\mu }\right)\sum_{int}\delta (t-{t}_{k-int}-{\tau }_{L})\right)\end{array}$$

12 $$\begin{array}{c}{\tau }_{rA}\frac{{dx}_{Ak}}{dt}={-x}_{Ak}+{\tau }_{m}\left(\begin{array}{c}{e}^{\frac{-r}{D}}\left({J}_{k-pyr}+{{e}^{\frac{r}{{D}_{s}}} \eta }_{IN}\left({x}_{pre}-{x}_{tar}\right){\left({w}_{max}-w\right)}^{\mu }\right)\sum_{pyr}\delta (t-{t}_{k-pyr}-{\tau }_{L})\\ +{J}_{k-ext}\sum_{ext}\delta (t-{t}_{k-ext}-{\tau }_{L})\end{array}\right)\end{array}$$

13 $$\begin{array}{c}{\tau }_{rG}\frac{{dx}_{Gk}}{dt}={-x}_{Gk}+{\tau }_{m}\left({e}^{\frac{-r}{D}}\left({J}_{k-int}-{{\text{e}}}^{\frac{{\text{r}}}{{{\text{D}}}_{{\text{s}}}}} {\eta }_{IN}\left({x}_{pre}-{x}_{tar}\right){\left({w}_{max}-w\right)}^{\mu }\right)\sum_{int}\delta (t-{t}_{k-int}-{\tau }_{L})\right)\end{array}$$

As the spike rate in the pattern classification network rises, the learning speed also increases, which means that interneurons release fewer neurotransmitters into the synaptic cleft compared to pyramidal neurons (${\eta }_{IN}=0.5 {\eta }_{PY}$).

The chosen STDP rule, which employs a power-law function, delivers comparable classification accuracies compared to other STDP variants using the exponential functions described in previous studies. It's worth noting that the power-law weight-dependent STDP rule offers the advantage of triggering weight updates only when a post-synaptic pyramidal neuron or interneuron fires a spike. Given the relatively low firing rate of post-synaptic neurons, this more intricate STDP update mechanism does not demand significant computational resources. Previous research has demonstrated that the power-law weight dependence of the STDP learning rule enhances learning robustness and accelerates convergence^29^.

## CORDIC based computation: Exp CORDIC, Ln CORDIC, and Pow CORDIC

The CORDIC algorithm represents an iterative computational technique applied to calculate a variety of complex functions, encompassing multiplication, exponentials, logarithms, hyperbolic, and trigonometric functions. This method operates efficiently by utilizing simple shift and add operations while avoiding resource-intensive and slow arithmetic multipliers. Consequently, CORDIC functions can be readily and effectively implemented in digital Application-Specific Integrated Circuits (ASICs) and FPGAs^39^. Consequently, CORDIC tends to outperform alternative methods in scenarios where a hardware multiplier is unavailable, such as in microcontroller platforms, or when minimizing gate usage is a critical factor, as in FPGA or ASIC implementations. CORDIC offers a highly precise and cost-effective means of implementing nonlinear dynamic properties like natural exponentials and power-law functions in the context of most biologically plausible SNN models and STDP learning rules, such as the chosen SNN and Spatial-Pow-STDP. This paper introduces novel algorithms of CORDIC tailored to natural exponentials (Exp CORDIC), natural logarithms (Ln CORDIC), and arbitrary power-law (Pow CORDIC) functions. Subsequently, it evaluates and compares their accuracy and computational iterations performance, considering the specific network model and learning rule employed.

The section introduces the hyperbolic CORDIC, forming the foundation for the advanced Exp CORDIC, Ln CORDIC, and Pow CORDIC algorithms. Following this, the algorithms are presented and elaborated upon in detail. Subsequently, a comprehensive evaluation and comparison are conducted with similar methodologies introduced in existing literature.

### Introduction to hyperbolic CORDIC

The iterative formula of the fundamental version of hyperbolic CORDIC is provided as follows^24^:

14a $$\begin{array}{c}{x}_{i+1}={x}_{i}+{\sigma }_{i}{2}^{-i}{y}_{i}\end{array}$$

14b $$\begin{array}{c}{y}_{i+1}={y}_{i}+{\sigma }_{i}{2}^{-i}{x}_{i}\end{array}$$

14c $$\begin{array}{c}{z}_{i+1}={z}_{i}-{\sigma }_{i}{{\text{tanh}}}^{-1}\left({2}^{-i}\right)\end{array},$$

where *i* is an integer commencing at 1, the values of ${\sigma }_{i}$ can be established according to the chosen mode of operation. Hyperbolic CORDIC is categorized into two distinct modes: rotation mode CORDIC and vectoring mode CORDIC. In the Rotation mode of Hyperbolic CORDIC (RH CORDIC), the ${\sigma }_{i}$ values are determined as ${\sigma }_{i}=sign\left({z}_{i}\right)$, whereas in the Vectoring mode of Hyperbolic CORDIC (VH CORDIC), the ${\sigma }_{i}$ values are designated as ${\sigma }_{i}=-sign({z}_{i})$.

After completing the iterations, *z* is driven to 0 by RH CORDIC, while *y* is driven to 0 by VH CORDIC. It is imperative to acknowledge in hyperbolic CORDIC that when the iterative sequence number *i* corresponds to 4, 13, 40 …, *K*, 3*K* + 1, the respective iteration stage must be executed twice to ensure convergence. After numerous iterations, the outputs of RH CORDIC and VH CORDIC will ultimately converge to specific results, as expressed by the following equations, respectively:

15 $$\begin{array}{c}RH\, CORDIC\to \left\{\begin{array}{c}{x}_{n}={K}_{H}({x}_{0}cosh{z}_{0}+{y}_{0}sinh{z}_{0})\\ {y}_{n}={K}_{H}({y}_{0}cosh{z}_{0}+{x}_{0}sinh{z}_{0})\\ {z}_{n}=0\end{array}\right.\end{array}$$

16 $$\begin{array}{c}VH\, CORDIC\to \left\{\begin{array}{c}{x}_{n}={K}_{H}\sqrt{{x}_{0}^{2}-{y}_{0}^{2}}\\ {y}_{n}=0\\ {z}_{n}={z}_{0}+{{\text{tanh}}}^{-1}\left({y}_{0}/{x}_{0}\right)\end{array}\right.\end{array}$$

where scale factor ${K}_{H}$ for hyperbolic CORDIC can be determined through the following computation:

17 $$\begin{array}{c}{K}_{H}= \prod_{i=1}^{n}\left(\sqrt{1-{2}^{-2i}}\right).\end{array}$$

It should be noted that in Eq. (17), elements related to repeated iterations are to be multiplied twice. Equations (15) and (16) demonstrate that hyperbolic CORDIC can calculate inverse hyperbolic tangent, hyperbolic sine, and hyperbolic cosine. This capability forms the basis for computing natural exponentials and natural logarithms, as elucidated in prior publications^39,40^.

In a more detailed manner, for the computation of natural exponentials, the initialization of the input for RH CORDIC is defined as ${x}_{0}=1/{K}_{H}$, ${y}_{0}=0$, and ${z}_{0}=R$. Consequently, as per Eq. (15), the results of this operation can be formulated as ${x}_{n}=cosh\left(R\right)$ and ${y}_{n}=sinh\left(R\right)$. Consequently, natural exponentials are computed by performing an addition operation between these outputs, which is calculated as follows:

18 $$\begin{array}{c}{x}_{n}+{y}_{n}={\text{cosh}}\left(R\right)+{\text{sinh}}\left(R\right)={\text{exp}}\left(R\right).\end{array}$$

Additionally, to compute natural logarithms, it is necessary to initialize the inputs of VH CORDIC as ${x}_{0}=R+1$, ${y}_{0}=R-1$, and ${z}_{0}=0$. Subsequently, as per Eq. (16), the resulting output of VH CORDIC, denoted as ${z}_{n}$, can be expressed as follows:

19 $$\begin{array}{c}{z}_{n}={{\text{tanh}}}^{-1}\left(\frac{R-1}{R+1}\right)=\frac{1}{2}{\text{ln}}\left(R\right)\end{array}$$

the actual value of ln(*R*) can be obtained by left-shifting ${z}_{n}$ by one bit to multiply it by 2.

Hence, the CORDIC algorithm can calculate exp(R) and ln(R). Utilizing these values, power-law functions with arbitrary exponents can also be determined and computed with CORDIC as follows:

20 $$\begin{array}{c}{x}^{p}={\text{exp}}\left(p\times {\text{ln}}\left(x\right)\right).\end{array}$$

Up to this point, the method for deriving natural exponentials and natural logarithms through hyperbolic CORDIC has been elucidated, followed by the computation of power-law functions using the same technique. Advanced algorithms, namely Exp CORDIC, Ln CORDIC, and Pow CORDIC, have been introduced based on Eqs. (18), (19), and (20) to calculate natural exponentials, natural logarithms, and arbitrary power-law functions, respectively.

### Proposed Exp CORDIC, Ln CORDIC, and Pow CORDIC algorithms

Previous studies^39–44^ have indicated that the traditional CORDIC algorithm suffers from a significant limitation of slow convergence, requiring redundant iterations that can account for up to 50% of the total iterations before reaching the desired target angle. Furthermore, other algorithms proposed in prior research have exhibited inadequate convergence speed and accuracy^39–44^, essential attributes for implementing SNNs and associated learning rules in digital ASIC and FPGA platforms. In light of these challenges, this paper introduces novel CORDIC algorithms aimed at achieving faster and more precise convergence to the final target with the fewest elementary iterations, addressing these issues.

The Exp CORDIC algorithm and its requisite components, is illustrated in Algorithm 1.

**Algorithm 1:**
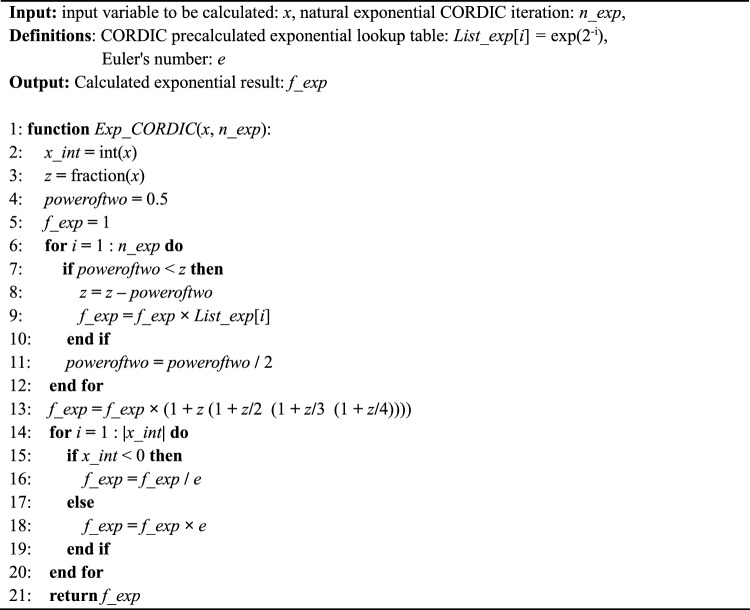
CORDIC for calculating natural exponentiation: ***Exp CORDIC***.

In Exp CORDIC, pre-computed values of exp(2^–1^), exp(2^–2^), exp(2^–3^), …, exp(2^−n^) are stored in an array denoted as *List_exp*[*i*]. In line 13, the *Maclaurin* series definition is utilized to enhance accuracy. Additionally, the parameter *n_exp*, representing the number of iterations employed in hyperbolic CORDIC, determines the closeness of the result *f_exp* to the actual natural exponential value, albeit at the expense of latency and implementation resources. The proposed algorithm does not limit the input range *x*; however, it necessitates a sufficiently high number of iterations. Depending on the input range *x* and its integer part *x_int*, the *IF* condition in line 15 or its *ELSE* counterpart in line 17 can be applied accordingly.

**Figure 5:**
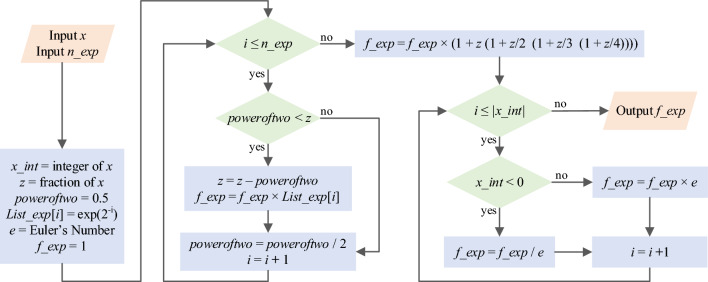
Computing flow of proposed Exp CORDIC approach for calculating exp(*x*) with *n_exp* iteration.

Furthermore, the computation process of the Exp CORDIC is depicted in Fig. 5 based on its algorithm. Notably, all functions within Exp CORDIC can be executed using shift, add, and subtraction operations, obviating the need for a multiplier. This innovative algorithm demonstrates swifter convergence compared to algorithms introduced in subsequent publications. Nonetheless, a comprehensive performance analysis and comparison of the proposed algorithm are presented in the subsequent subsection.

Moreover, the proposed Ln CORDIC and its requisite components, is shown in Algorithm 2.

**Algorithm 2:**
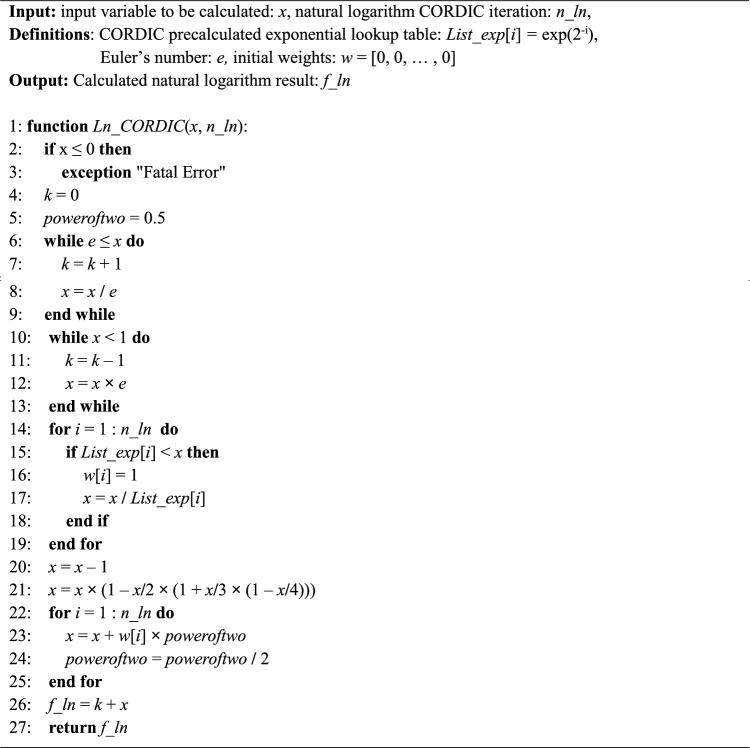
CORDIC for calculating natural logarithm: ***Ln CORDIC***.

**Figure 6:**
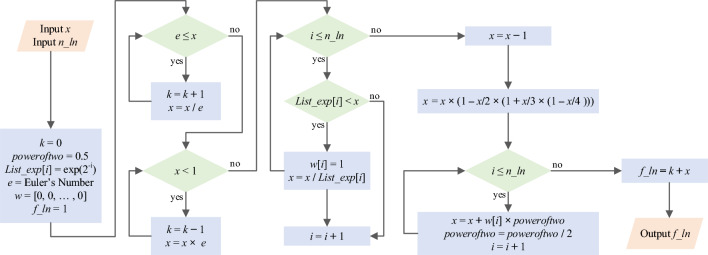
Computing flow of proposed Ln CORDIC algorithm for calculating ln(*x*) with *n_ln* iteration.

In Ln CORDIC, precomputed values of exp(2^–1^), exp(2^–2^), exp(2^–3^), …, exp(2^−n^) are stored in an array denoted as *List_exp*[*i*]. In line 21, the *Maclaurin* series definition is applied to enhance accuracy. Additionally, the parameter *n_ln*, representing the number of iterations employed in hyperbolic CORDIC, influences the proximity of the result *f_ln* to the actual natural logarithm value, albeit at the expense of latency and implementation resources. The input range *x* in the proposed algorithm only needs to be above zero; otherwise, an exception occurs. The number of iterations must be sufficiently high for a broader range of inputs. Depending on the input range *x*, one of the *WHILE* loops in lines 6 or 10, or none of them, can be utilized. In addition, the Ln CORDIC computing flow is illustrated in Fig. 6, following its algorithm. Notably, all functions utilized in Ln CORDIC can be executed using shift, add, and subtraction operations, eliminating the need for a multiplier. This state-of-the-art algorithm is the fastest and most accurate method for calculating natural logarithms using CORDIC. However, a detailed performance analysis and comparison of the proposed algorithm are presented in the subsequent subsection.

Thus far, two CORDIC algorithms have been introduced for the computation of natural exponentials and natural logarithms. Subsequently, in accordance with Eq. (20), power-law functions incorporating arbitrary exponent terms can be computed using the CORDIC concept. Consequently, leveraging the foundation provided by Exp CORDIC and Ln CORDIC, the Pow CORDIC algorithm and its constituent elements are presented in Algorithm 3.

**Algorithm 3:**
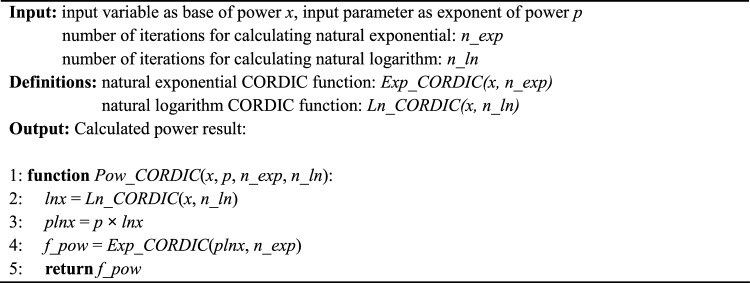
CORDIC for calculating arbitrary power-law function: ***Pow CORDIC***.

**Figure 7:**
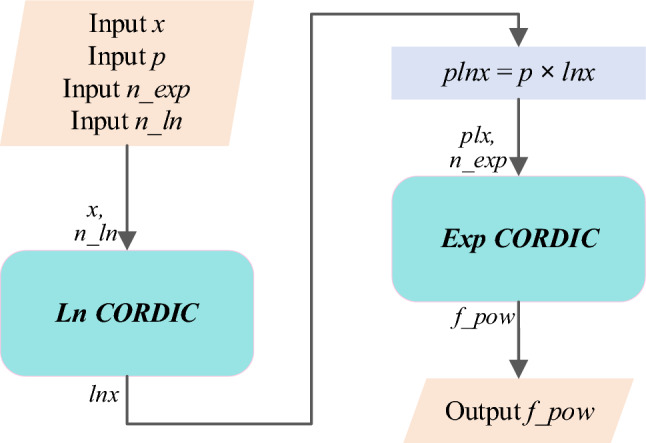
Computing flow of suggested Pow CORDIC approach for finding *x*^*p*^ with fixed arbitrary *p* exponent using Exp CORDIC and Ln CORDIC and their iterations. All internal multiplications and divisions are implemented using only shift and addition operations.

All the components of Pow CORDIC are presented in Algorithms 1 and 2. Here, *p* signifies the arbitrary exponent selected for raising the input *x* to the power of *p*. Figure 7 depicts computing following the suggested Pow CORDIC approach. Since the exponent *p* is predetermined and unchanging, the multiplication operation in line 3, alongside other functions, can be executed through shift and add operations. This proposed CORDIC exhibits swift and precise convergence due to its principled approach and the utilization of efficient and accurate functions. Nonetheless, the performance of Pow CORDIC is subjected to analysis and comparison in the subsequent subsection.

### Performance analysis of the proposed CORDIC algorithms

In this sub-section, the errors of various iterations in different CORDIC simulations are examined and analyzed to assess the accuracy of CORDIC-based operations in the SNN network and the learning rule for classification. The proposed methodologies are based on iterative Algorithms 1, 2, and 3, as well as the top-level computing flow depicted in Figs. 1, 2, and 3, have been implemented and coded. To evaluate the accuracy, the average mean of relative error has been chosen as the representative measure. The average mean of relative error is defined as follows:

21 $$\begin{array}{c}Avg Err=\frac{{\sum }_{i=0}^{Num}\left|\frac{T-C}{T}\right|}{Num}.\end{array}$$

In Eq. (21), *T* signifies the true values of exponential, logarithmic, or power-law functions obtained using Python's libraries. *C* denotes the computed outcomes derived from the proposed methodology, while *Num* represents the count of calculated samples for each respective function.

The proposed Exp CORDIC algorithm for computing natural exponential was initially subjected to testing. Table 1 displays a comprehensive performance analysis, including error comparison, across various CORDIC methods utilized for natural exponentiation computation and implementation. This analysis encompassed 1 million random inputs assessed through a specific number of iterations. To ensure a fair comparison, the input range for each method was specified. However, it is worth noting that the proposed algorithm in this paper for exponential computation does not possess a limited range, as previously explained.

**Table 1:** Exp CORDIC average error analysis and comparison based on input range and number of iterations.

CORDICs for exp	input range	*n_exp* = 2	*n_exp* = 4	*n_exp* = 8	*n_exp* = 10	*n_exp* = 12
conventional CORDIC (Eq. (18))	$[-1.1182 , 1.1182]$	$1.28\times {10}^{-1}$	$3.37\times {10}^{-2}$	$2.99\times {10}^{-3}$	$1.45\times {10}^{-3}$	$1.06\times {10}^{-3}$
Heidarpur's CORDIC^42^	$[0 , 1]$	$1.15\times {10}^{-1}$	$3.07\times {10}^{-2}$	$1.95\times {10}^{-3}$	$4.89\times {10}^{-4}$	$1.22\times {10}^{-4}$
Wu's CORDIC^41^	$[-1 , 1]$	$4.21\times {10}^{-2}$(n = 2.27)	$1.05\times {10}^{-2}$(n = 4.18)	$8.51\times {10}^{-5}$(n = 7.88)	$4.42\times {10}^{-7}$(n = 10.48)	–
Luo’s CORDIC^40^	$[-1.1178 , 1.1178]$	–	–	–	–	$1.2228\times {10}^{-4}$
Mopuri's CORDIC^44^	$[-6.9263 , 6.9263]$	–	$3.01\times {10}^{-2}$	$2\times {10}^{-3}$	–	$1.2224\times {10}^{-4}$
Exp CORDIC (Proposed)	$\left[-700 , 700\right] (-\infty , +\infty )$	$1.14\times {10}^{-6}$	$1.26\times {10}^{-9}$	$2.88\times {10}^{-14}$	$2.95\times {10}^{-14}$	$2.94\times {10}^{-14}$

Nevertheless, for testing, the input range was set between − 700 and 700 based on simulation limitations. The impact of the number of iterations (denoted as *n_exp*) on the results was of particular interest. Specific iterations were chosen as benchmarks, considering the pivotal role that the iteration count plays. As mentioned earlier, an increase in iterations significantly reduces average error, albeit at the cost of heightened latency and resource requirements. The results demonstrated that the Exp CORDIC method exhibited the smallest error from all perspectives. Notably, it achieved an average error of approximately 10^–14^ in computing natural exponential with just 12 iterations, significantly outperforming other methods. Consequently, the Exp CORDIC algorithm is exceptionally well-suited for implementing SNN networks, learning rules, and dynamic functions involving natural exponential computations.

Subsequently, the proposed Ln CORDIC algorithm for natural logarithm computation underwent testing. Table 2 presented a comprehensive performance analysis and facilitated comparison among different CORDIC methods employed for natural logarithm computation and implementation. This analysis encompassed 1 million random inputs, evaluated through a specified number of iterations. The input range for each method was detailed to ensure a meaningful comparison. However, it is noteworthy that the proposed algorithm for natural logarithm computation presented in this paper does not possess any range limitations; therefore, it can extend well beyond zero.

**Table 2:** Ln CORDIC average error analysis and comparison based on input range and number of iterations.

CORDICs for ln	input range	*n_ln* = 2	*n_ln* = 4	*n_ln* = 8	*n_ln* = 10	*n_ln* = 12
conventional (Eq. 19)	$[0.1068 , 9.3595]$	$1.72\times {10}^{0}$	$5.61\times {10}^{-1}$	$2.71\times {10}^{-1}$	$2.16\times {10}^{-1}$	$2.58\times {10}^{-1}$
Luo’s CORDIC^40^	$[0.107 , 9.352]$	–	–	–	–	$3.8023\times {10}^{-4}$
Mopuri's CORDIC^44^	$[9.6358\times {10}^{-7} , 1.0378\times {10}^{6}]$	–	$4.7\times {10}^{-3}$	$3.0735\times {10}^{-4}$	–	$1.923\times {10}^{-5}$
Chen's CORDIC^43^	$[6.3471\times {10}^{-8} , 6.8351\times {10}^{4}]$	–	–	–	–	$6.35\times {10}^{-5}$
Ln CORDIC (Proposed)	$\left(0 , {10}^{9}\right] (0 ,+\infty )$	$1.21\times {10}^{-4}$	$1.61\times {10}^{-6}$	$3.81\times {10}^{-10}$	$5.93\times {10}^{-12}$	$9.37\times {10}^{-14}$

Nevertheless, for testing, the input range was constrained to fall between 0 and 10^9^, dictated by simulation constraints. The number of iterations, denoted as *n_ln*, was observed to significantly influence the results. Specific iterations were singled out as benchmarks for analysis. As previously mentioned, an increase in the number of iterations led to a substantial reduction in average error, though at the expense of increased latency and resource utilization. The findings revealed that the Ln CORDIC method consistently exhibited the smallest errors from all perspectives. Notably, it achieved an average error of approximately 10^–14^ in natural logarithm computation with just 12 iterations, significantly outperforming other methods. Therefore, the Ln CORDIC algorithm is well-suited for implementing SNN networks, learning rules, and dynamic functions involving natural logarithmic operations.

The error analysis of the Pow CORDIC method is finally conducted and presented in Table 3. The Pow CORDIC approach is employed for calculating the power of a random input with a fixed, user-defined exponent. This method utilizes Ln CORDIC and Exp CORDIC, each with selectable number of iterations. Given that the computation of natural logarithms necessitates a higher number of iterations, a correspondingly elevated iteration count was adopted. Additionally, 1 million inputs were utilized to encompass the maximum achievable simulation range, although it should be noted that the method itself does not possess a restricted range. Considering that, in the context of the learning rule Eq. (8), the base of the power-law function consistently falls within the range of 0 to 1, a separate error analysis is conducted specifically for this interval. Moreover, Eq. (8) prescribes an exponent, denoted as *μ*, constrained to the range of 0 to 1. For this analysis, the exemplar exponent is denoted as *p* and varies between 0 and 1. The results in Table 3 demonstrate that the Pow CORDIC method can achieve an exceptionally low average error, approximately on the order of 10^9^, with just 4 and 8 iterations for exponential and natural logarithmic components, respectively.

**Table 3:** Pow CORDIC average error analysis based on input range and different iterations for exp and ln.

CORDIC for pow	input *x* range	*n_exp* = 2 *n_ln* = 4	*n_exp* = 4 *n_ln* = 5	*n_exp* = 4 *n_ln* = 8
Pow CORDIC	$(0 , 1)$	$1.78\times {10}^{-5}$	$1.97\times {10}^{-6}$	$5.1\times {10}^{-9}$
*p* in range (0, 1) (Proposed)	$\left(0 , 700\right)(0 , +\infty )$	$1.7\times {10}^{-5}$	$1.94\times {10}^{-6}$	$5\times {10}^{-9}$

In conclusion, the average error analyses and comparisons have demonstrated the precision and efficiency of the Pow CORDIC, Ln CORDIC, and Exp CORDIC algorithms. These algorithms are highly accurate and swift, rendering them suitable for integration within various aspects of SNN network functions, learning processes, or other applications where timely and precise results are essential. Furthermore, this approach offers the advantage of implementing these functions using solely shift and addition operations.

CORDIC algorithms are used in the network learning module, and the error in this module causes the change of network weights to be affected during the SNN learning process. This effect is such that if the accuracy of the output of these calculations is not at a good level, the learning of the network is done with less accuracy, and the speed of convergence of learning is reduced. Since accuracy and speed in the learning module are important to us, the Exp and Ln CORDIC iterative calculations are set to 4–5 to provide the required high accuracy. As a result, it provides high accuracy in the performance of the classification network and increases the convergence speed of network learning.

## Hardware design and implementation

### CORDIC-based hardware design

In addition to the CORDIC design and the evaluation carried out through theoretical analysis and algorithm definition in the preceding section, it is imperative to undertake hardware design and implementation to assess the effectiveness of the proposed CORDIC calculations. Given the advantages of flexibility, reconfigurability, and extensive parallel processing capabilities of FPGAs, this research endeavors to utilize a Xilinx Zynq FPGA device (xc7z030fbg484-3). The objective is to implement CORDIC algorithms as blocks using the VHDL programming language in the ISE Design Suite 14.7 environment, enabling the thorough examination of hardware implementation efficiency and accuracy across all of the proposed algorithms and the learning mechanism.

#### CORDIC algorithms implementation design

In this section, a comprehensive elaboration is provided on the design aspects of each algorithm. The primary objective is to optimize the utilization of resources in the implementation process while concurrently achieving the utmost accuracy in computationally demanding hardware tasks. In light of the prerequisite for a predefined and unchanging hardware architecture, the choice of iterations is guided by carefully considering the results delineated in Tables 1, 2, and 3. As a result, a selection of 4 and 5 iterations is made for the Exp and Ln CORDICs, respectively, to ensure an appropriate level of accuracy. Consequently, in the case of the Pow CORDIC, a cumulative total of 9 iterations is employed to facilitate the computation of arbitrary power functions.

In order to achieve a highly accurate and efficient implementation of Exp CORDIC, it is appropriate to allocate 8 fractional and 3 integral bits. This configuration enables the accommodation of input values up to 8, with a resolution step of 0.0039, while maintaining an average error of approximately ${10}^{-3}$. It should be noted that the hardware design is done according to the specific application and characteristics of the neural network learning block. When the accuracy of the required calculations, the input range, and the output result are known, as a result, we can determine the number of iterations and the number of bits in these specific conditions to avoid overflow and underflow. Consequently, an 11-bit representation is employed for Exp CORDIC, accounting for potential overflow and underflow conditions. However, a greater bit count may be warranted for applications necessitating extended input ranges and enhanced resolution. Moreover, since the input of the Exp CORDIC algorithm is derived from the Ln output, it is characterized by values exclusively below 0. Consequently, during the implementation, certain superfluous conditions in the Exp CORDIC algorithm, such as those found in line 17 of Algorithm 1, can be omitted, reducing hardware resource utilization.

**Figure 8:**
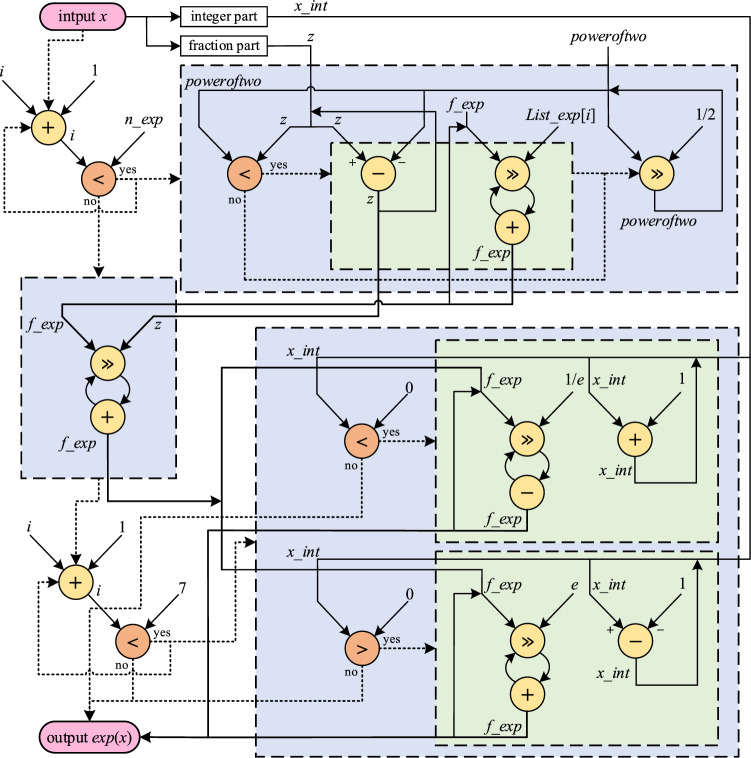
Implementation architecture of Exp CORDIC for *n_exp* iteration. All the solid lines are for data transition and dotted ones are control lines for enabling or disabling. For each loop, *i* starts at 0, and other parameter initializations are based on Algorithm 1.

The engineered architecture for the implementation of Exp CORDIC is illustrated in Fig. 8. Multiplications are executed through right shifting and addition, while deviations are carried out through right shifting and subtraction. Non-constant values, such as *x_int* and *z*, are implemented as fixed values and linear approximations proportional to the input. Moreover, to minimize resource costs while maintaining the desired accuracy, the *Maclaurin* series in line 13 of Algorithm 1 is simplified to its first component.

For the achievement of a sufficient accuracy and efficient implementation of Ln CORDIC, 12 fractional and 3 integral bits were assigned, resulting in a permissible input and output range spanning from 0.000244 to nearly 8, which yields an average error of approximately ${10}^{-4}$. Additionally, a single bit was employed as a sign bit to handle negative natural logarithm results. Thus, in this paper's application, 16 bits are appropriately employed for Ln CORDIC, considering the potential for overflow and underflow. A greater number of bits can be employed for applications with wider input ranges or requiring higher resolution. As the natural logarithm input in our adopted learning mechanism is represented as $({w}_{max}- w)$, the input values fall exclusively within the range of 0 to 1, and any conditions within the algorithm that pertain to values outside this range remain unimplemented, akin to the exclusion of line 6 in Algorithm 2. Furthermore, attention should be directed to the computation series defined in line 21 of Algorithm 2, partially implemented in this application, extending up to a power of two. To achieve the requisite accuracy commensurate with Ln CORDIC precision, the power of two computations is accomplished using Square CORDIC, as presented in a prior publication^24^, employing the same bit width and requiring 12 iterations.

**Figure 9:**
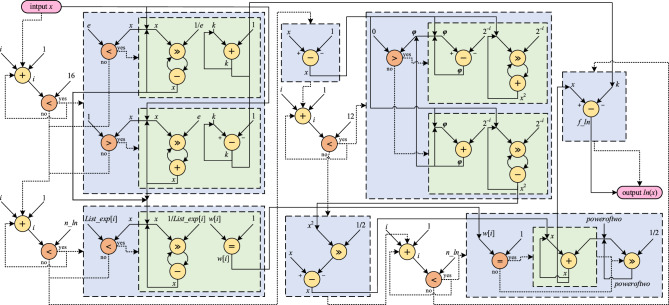
Implementation architecture of Ln CORDIC for *n_ln* iteration. All the solid lines are for data transition and dotted ones are control lines for enabling or disabling. For each loop, *i* starts at 0 and other parameter initializations are based on Algorithm 2.

The configured architecture for the implementation of Ln CORDIC is depicted in Fig. 9. Multiplications are executed through right shifting and addition, while deviations are achieved through right shifting and subtraction. The computation of Ln involves utilizing a *Maclaurin* series, as indicated in Algorithm 2 line 21, with the square component employed for efficient and precise implementation. The Square CORDIC, introduced in a prior paper^24^ for computing *x*^*2*^ through 12 iterations, is utilized for the square function. Additionally, non-constant operations within the *WHILE* loop are implemented as values proportional to the input.

**Figure 10:**
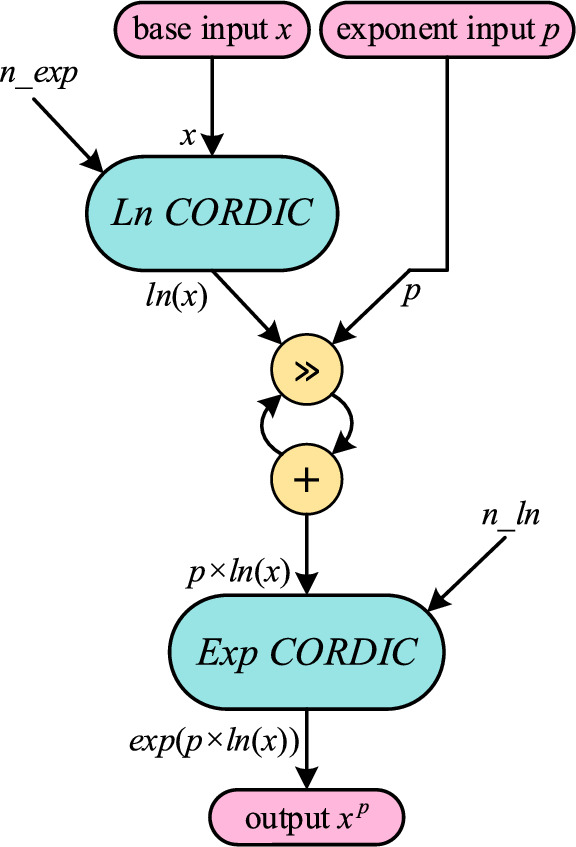
Implementation architecture of Pow CORDIC.

In the context of Pow CORDIC, which comprises components from the preceding explanations, a 16-bit allocation is applied for the computation of arbitrary power functions with a predetermined fixed exponent, resulting in a target error level of approximately ${10}^{-3}$. It is evident that to achieve even lower error margins, a greater bit width may be chosen, and the selection of additional CORDIC iterations is an option. However, it is noteworthy that within the specific context of this paper, which focuses on applying a particular learning mechanism and SNN architecture, the designated CORDIC designs yield highly favorable outcomes, as elaborated upon in the Results Section. It is important to emphasize that all these designs operate in parallel mode, delivering results within a single clock cycle to ensure rapid computation, although this approach entails increasing resource consumption. As shown in Fig. 10, Pow CORDIC can be implemented using Ln and Exp CORDIC blocks plus multiplication, which is implemented using right shift and addition to compute *x*^*p*^*.*

#### Learning block implementation design

**Figure 11:**
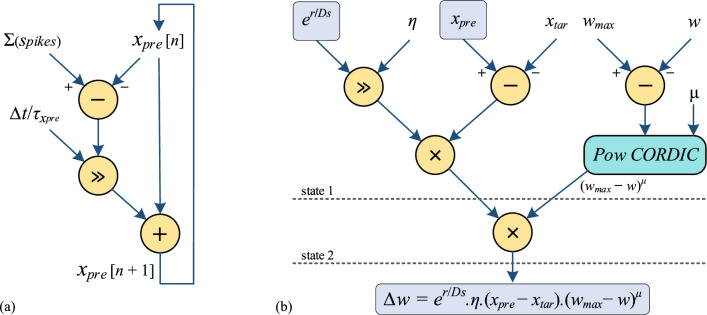
Data flow of (**a**) pre-synaptic trace named ${x}_{pre}$ (**b**) Spatial-Pow-STDP learning. In the learning block, the amount of weight change is measured in 2 states using only one repeated multiplier.

In this section, a comprehensive design detailing the data flow of the learning process is presented to ensure an efficient implementation. Figure 11 illustrates the scheduling diagram for computing $\Delta w$ and ${x}_{pre}$, as introduced in Eqs. (8) and (9), respectively. The fixed-point hardware architecture for these computations comprises 1 sign bit, 3 integral bits, and 12 fractional bits, facilitating the attainment of the required accuracy and enabling an efficient implementation.

To implement ${x}_{pre}$, the differential equation within its definition can be discretized through the straightforward application of Euler's method. This approach generates stable output results by selecting small step sizes to ensure the stability of the Euler method. As depicted in Fig. 11a, in order to compute the subsequent value of ${x}_{pre}$ denoted as ${x}_{pre}[n + 1]$, the initial value of ${x}_{pre}$ denoted as ${x}_{pre}[n]$ is first subtracted from the summation of spikes generated by all pre-synaptic neurons. The outcome of this subtraction is then multiplied by a constant through a shift operation. Subsequently, the result of the shift operation is added to ${x}_{pre}[n]$ to determine the desired result, ${x}_{pre}[n + 1]$.

Furthermore, in the computation of $\Delta w$, there is a requirement for ${x}_{pre}$ and ${e}^\frac{r}{Ds}$, which are acquired, respectively, from the detailed ${x}_{pre}$ scheduling diagram and the Exp CORDIC using the input value $\frac{r}{Ds}$. Multiplication by the constant learning rate $\eta$ is achieved through a basic shift operation. The complete data flow for $\Delta w$ is visually represented in Fig. 11b, comprising a Finite State Machine (FSM) with two distinct states and a Pow CORDIC block employed to calculate the power of $\mu$ with 9 iterations. Two multiplicative operations are employed in the formulation of Spatial-Pow-STDP for ascertaining the extent of weight adjustment during the learning process. To streamline this process into a single multiplication, two sequential multipliers are implemented within the serial states of a simplified FSM. This approach facilitates the determination of $\Delta w$ within two clock cycles.

### FPGA implementation analysis

In this part, the designs that were explicated in the previous part have been realized through VHDL implementation in Xilinx's Zynq FPGA device xc7z030fbg484-3 using ISE Design Suite 14.7. The implementation results for the proposed methods are presented in Tables 4, 5, and 6. It is noteworthy that the bold method is employed in the Spatial-Pow-STDP and SNN. The average error for each implementation is calculated using Eq. (21), with 5 million random samples. The input range for Exp ranges from –7 to 0, and for Ln, it spans from 0 to 1. The low average error contributes to high learning accuracy, leading to superior classification accuracy. The exceptional maximum speed also allows a rapid learning mechanism, meeting a critical requirement in neural networks.

**Table 4:** Hardware utilization, maximum speed, and average error of proposed Exp CORDIC implementations for different numbers of *n_exp* iterations.

Implementation in 11-bits	LUT	Register slice	DSP	Max speed	Average error
Exp CORDIC with 2 iterations	330	0	0	768 MHz	6.76 × 10^–2^
Exp CORDIC with 3 iterations	370	0	0	769 MHz	1.83 × 10^–2^
**Exp CORDIC with 4 iterations**	**370**	**0**	**0**	**769 MHz**	**5.67 × 10**^**–3**^
Exp CORDIC with 5 iterations	326	0	0	768 MHz	5.63 × 10^–3^
Exp CORDIC with 6 iterations	330	0	0	685 MHz	6.44 × 10^–3^
Exp CORDIC with 7 iterations	349	0	0	685 MHz	1.00 × 10^–2^

The bold one is the selected architecture.

**Table 5:** Hardware utilization, maximum speed, and average error of proposed Ln CORDIC implementations for different numbers of *n_ln* iterations.

Implementation in 16-bits	LUT	Register slice	DSP	Max speed	Average error
Ln CORDIC with 2 iterations	2182	289	0	771 MHz	9.59 × 10^–2^
Ln CORDIC with 3 iterations	2250	301	0	771 MHz	8.94 × 10^–3^
Ln CORDIC with 4 iterations	2321	310	0	771 MHz	7.49 × 10^–4^
**Ln CORDIC with 5 iterations**	**2393**	**320**	**0**	**771 MHz**	**4.09 × 10**^**–4**^
Ln CORDIC with 6 iterations	2482	331	0	771 MHz	6.05 × 10^–4^
Ln CORDIC with 7 iterations	2564	340	0	771 MHz	5.17 × 10^–4^

The bold one is the selected architecture.

**Table 6:** Hardware utilization, maximum speed, and average error of proposed Pow CORDIC implementations for different numbers of *n_exp* and *n_ln* iterations as natural exponential and natural logarithm.

Implementation in 16-bits	LUT	Register slice	DSP	Max speed	Average error
Pow CORDIC with 3 Exp iterations and 3 Ln iterations	2699	305	0	685 MHz	2.21 × 10^–2^
Pow CORDIC with 3 Exp iterations and 4 Ln iterations	2773	313	0	767 MHz	2.21 × 10^–2^
Pow CORDIC with 4 Exp iterations and 4 Ln iterations	2804	314	0	685 MHz	6.94 × 10^–3^
**Pow CORDIC with 4 Exp iterations and 5 Ln iterations**	**2876**	**324**	**0**	**685 MHz**	**6.93 × 10**^**–3**^
Pow CORDIC with 5 Exp iterations and 5 Ln iterations	2915	323	0	685 MHz	5.87 × 10^–3^
Pow CORDIC with 5 Exp iterations and 6 Ln iterations	2999	334	0	685 MHz	5.87 × 10^–3^

The bold one is the selected architecture.

The average error in Tables 4 and 5 is due to the simplified hardware design of the Exp and Ln CORDIC blocks. These designs are optimized for specific applications and input ranges to achieve the required accuracy with a limited number of iterations. However, using these simplified designs with unoptimized iterations increases the average error. In contrast, Tables 1 and 2 show the results of the original algorithms without simplification, demonstrating that increasing the number of iterations decreases the average error, indicating higher accuracy. The average error of the original algorithms decreases steadily with more iterations until it reaches a saturation point, where it remains at about ${10}^{-14}$ for Exp and ${10}^{-15}$ for Ln for a wide range of input. This saturation is expected due to the approximate nature of the CORDIC algorithm compared to exact operators.

The outcomes include determining the maximum operating frequency and quantifying resource utilization, presented as a percentage of the available hardware resources on the selected FPGA device, as summarized in Table 7. The Spatial-Pow-STDP learning block, serving as a learning component in SNN, incorporates Pow CORDIC through 9 iterations, as depicted in the data flow illustrated in Fig. 11b. A superior operational speed of 776 MHz is demonstrated, coupled with a significantly low average error of 6.93 × 10^–3^. This performance underscores its effectiveness as a hardware computation solution with notable efficiency. Notably, the Spatial-Pow-STDP learning module predominantly consumes Look-Up Tables (LUTs), considered abundant resources due to the economization of relevant constants within the algorithms. Conversely, the utilization of Digital Signal Processors (DSPs), recognized as costly and relatively scarce resources in both FPGA and ASIC configurations, is minimal, with only one DSP employed to handle the multiplier operation within the Finite State Machine of the $\Delta w$ data flow. This choice ensures a straightforward and feasible implementation.

**Table 7:** Proposed CORDIC implementation result for power-law learning block.

Implementation	Slice LUTs (of 78,600)	Slice registers (of 157,200)	DSPs (of 400)	BRAM (of 530 18Kib)	Max speed	Average error
Exp CORDIC	370 (0.47%)	0	0	0	769 MHz	5.67 × 10^–3^
Ln CORDIC	2393 (3.04%)	320 (0.20%)	0	0	771 MHz	4.09 × 10^–4^
Pow CORDIC	2876 (3.65%)	324 (0.20%)	0	0	685 MHz	6.93 × 10^–3^
Spatial-Pow-STDP	2966 (3.77%)	357 (0.22%)	1 (0.25%)	0	776 MHz	6.93 × 10^–3^

The achieved average errors are primarily attributable to the inherent design characteristics of the proposed Exp CORDIC, Ln CORDIC, and Pow CORDIC algorithms, which can be effectively leveraged within neural network applications. It should be noted, however, that higher levels of precision can be attained by adopting a greater number of bits or additional iterations, as elucidated in the implementation design. The foundation of the proposed CORDIC algorithms lies in their precise ability to compute functions while maintaining hardware-friendliness. Since the classification network only has one learning block and the weight range is between 0 and 1, having low error and fast calculation is crucial, even at the expense of increased resource usage and power consumption.

Furthermore, the attained maximum speeds are notably elevated due to the utilization of designs structured in parallel architecture, albeit at the cost of increased resource utilization. The critical path in the proposed methodology resides in a shift-add operation, contrasting with the state-of-the-art method, where the critical path involves a multiplication operation. Generally, it is observed that the latency associated with multiplication exceeds that of a shift-add operation. Consequently, the proposed methodology exhibits the potential for a higher operating frequency. The presence of a DSP multiplier within the FPGA configuration diminishes the overall system frequency, thereby causing other system units to operate at a reduced pace. Conversely, the absence of a DSP component enhances the system speed, thereby augmenting overall system throughput.

The key advantage of CORDIC is substituting multiplications with fast shift and add operations. Therefore, for each specific multiplication, specific shift and add units are used instead. As repetitions increase, more unique shifts and adds modules are required to handle the repeated multiplications. This leads to higher overall hardware usage. In summary, extra hardware resources implement multiple CORDIC iterations and specialized shift/add units to optimize speed without compromising accuracy or delaying learning.

Table 8 presents the total power consumption of each CORDIC hardware design and the digital learning block. Since the CORDIC designs do not contain DSPs, the computations are energy-efficient. The total power consumption includes static and dynamic power per calculation performed in a single clock cycle. Using the XILINX XPower Analyzer tool in ISE Design Suite 14.7, the power usage can be measured at different speeds. This allows us to see how the energy usage increases at higher speeds.

**Table 8:** Proposed CORDIC implementations power consumption.

Total power consumption	Exp CORDIC	Ln CORDIC	Pow CORDIC	Spatial-Pow-STDP
Power (W) at max speed	0.14609 at 769 MHz	0.14621 at 771 MHz	0.14827 at 685 MHz	0.15548 at 776 MHz
Power (W) at a typical speed	0.14052 at 100 MHz	0.14054 at 100 MHz	0.14094 at 100 MHz	0.14173 at 100 MHz

## Results

In this section, the performance analysis and comparison of the proposed methodology are presented. The dynamics of pyramidal neurons, interneurons, and synapses in the neural network model align with the explanation provided in the Computational SNN Model section. A population of 5000 neurons and over 5 million synaptic connections in the spatiotemporal SNN undergo training through the discussed Spatial-Pow-STDP on the training data of MNIST, EMNIST, and CIFAR10 datasets. The learning process encompasses training synaptic weights for the second layer and the connections of the output layer.

To facilitate the attainment of a steady state for the variables in the dynamic equations, a resting time interval of 100 ms is interposed between two input patterns. The training of MNIST, EMNIST, and CIFAR10 classification networks involved 6, 2, and 6 epochs, respectively, until convergence. This decision stems from the observation that the performance accuracy on the test set does not exhibit a significant augmentation with an increased number of training epochs. Subsequently, the final training step in the image classification network involves the assignment of class labels to each neuron in the classifier layer (output layer) based on the one exhibiting the highest firing rate. Upon the conclusion of training, the synaptic weights undergo freezing. The mean accuracy on the respective test sets of datasets gauges the test performance of the trained networks. Network validation entails three training iterations, with accuracy results collected and averaged to provide the ultimate performance accuracy report.

In recent years, numerous spiking pattern classification networks have emerged. The MNIST, EMNIST, and CIFAR10 classification networks proposed in this study, when juxtaposed with preceding spiking pattern classification networks, demonstrate elevated accuracies even with reduced training epochs, thereby substantiating the superior efficacy of the proposed topology and learning approach. In contrast, the spiking pattern recognition networks trained through an unsupervised learning strategy exhibit comparatively diminished performance compared to deep networks trained with a supervised learning strategy. Conversely, the proposed network and learning approach manifest several noteworthy advantages over alternative networks, particularly deep networks:

1. A reduced number of hyper-parameters characterize the proposed network.
2. The proposed network is amenable to unsupervised training.
3. The proposed network operates on an event-based paradigm, lowering energy consumption.
4. Implementing the proposed network is viable on neuromorphic boards characterized by low power consumption.
5. The proposed network exhibits an accelerated convergence speed.

Finally, the test accuracies for the MNIST, EMNIST, and CIFAR10 datasets are compared with those of various recently introduced deep and spiking neural networks in Tables 9, 10, and 11, respectively. The proposed spiking image classification networks, encompassing both the software model and its hardware counterpart, exhibit higher classification accuracy in fewer training epochs when contrasted with several previously introduced spiking networks. This observation signifies an augmentation in the convergence rate facilitated by the proposed algorithms and architecture.

**Table 9:** Accuracy comparison with training epoch on MNIST dataset.

Network platform	Neural network	Learning mechanism	Learning method	Accuracy on MNIST (%)	Training epochs
Spiking	SNN^45^	Exponential STDP	Unsupervised	96.1	10
Spiking	SNN^46^	Exponential STDP	Unsupervised	97	8
Spiking	SNN^29^	Variants of STDP	Unsupervised	95	15
Spiking	Spatiotemporal SNN^27^	Spatial STDP	Unsupervised	97.3	8
Spiking	Spatiotemporal SNN	Spatial-Pow-STDP (Proposed)	Unsupervised	97.5	6
Spiking	Spatiotemporal SNN	CORDIC based Spatial-Pow-STDP (Proposed)	Unsupervised	97.47	6

**Table 10:** Accuracy comparison with training epoch on letters and digits of EMNIST dataset.

Network platform	Neural network	Learning mechanism	Learning method	Accuracy on EMNIST letters (%)	Accuracy on EMNIST digits (%)	Training epochs for letters	Training epochs for digits
Deep	CNN (Spinal FC)^47^	SpinalNet classification layers and transfer learning	Supervised	90.02	99.07	8	8
Deep	VGG-5 (Spinal FC)^48^	STDP	Supervised	95.79	99.75	200	50
Spiking	SNN using SpykeFlow^48^	STDP	Supervised	85.47	85.47	25	25
Spiking	Spatiotemporal SNN^27^	Spatial STDP	Unsupervised	93.1	97.45	3	3
Spiking	Spatiotemporal SNN	Spatial-Pow-STDP (Proposed)	Unsupervised	93.4	97.6	2	2
Spiking	Spatiotemporal SNN	CORDIC based Spatial-Pow-STDP (Proposed)	Unsupervised	93.4	97.59	2	2

**Table 11:** Accuracy comparison with training epoch on CIFAR10 dataset.

Network platform	Neural network	Learning mechanism	Learning method	Accuracy on CIFAR10 (%)	Training epochs
Spiking	SNN^46^	Exponential STDP	Unsupervised	92.4	8
Spiking	DIET-SNN^49^	Spike based back propagation	Supervised	92.7	< 20
Hybrid Deep and Spiking	ANN-SNN^50^	Spike-Norm	Supervised	91.55	30
Hybrid Deep and Spiking	SNN-Backprop^51^	Spike based back propagation	Supervised	91.41	150
Spiking	Spatiotemporal SNN^27^	Spatial STDP	Unsupervised	92.9	8
Spiking	Spatiotemporal SNN	Spatial-Pow-STDP (Proposed)	Unsupervised	93	6
Spiking	Spatiotemporal SNN	CORDIC based Spatial-Pow-STDP (Proposed)	Unsupervised	92.96	6

Superior accuracy is attained by the proposed network employing unsupervised learning in comparison to spiking networks derived from the conversion of deep neural networks into spiking counterparts. Although our classification demonstrates a lower accuracy than deep networks, this disparity can be contextualized by considering the heightened convergence speed and the aforementioned advantages inherent in the proposed networks. Regarding the representation of convergence time, since this parameter has not been presented in previous works, it is not possible to compare it.

**Figure 12:**
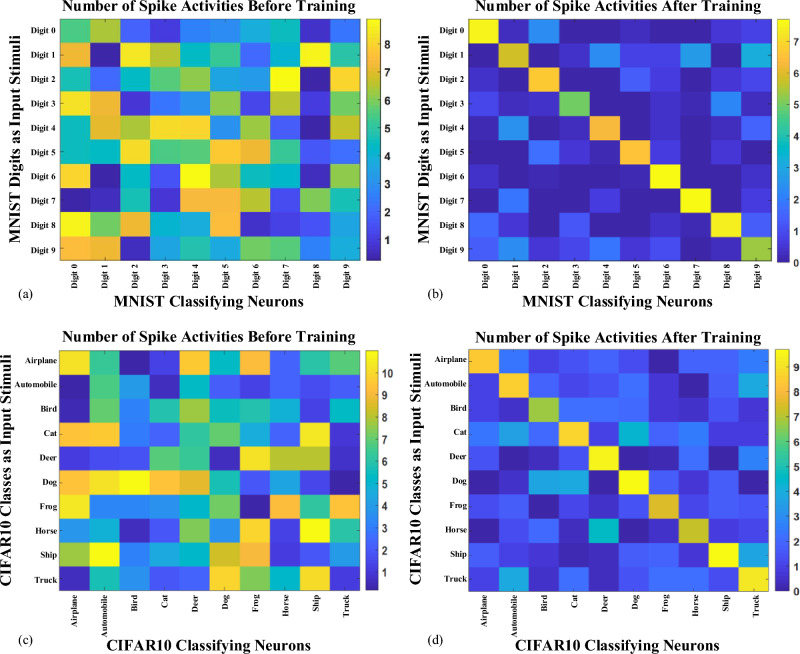
Correlation of spile activities. Figures (**a**) and (**b**) signify the count of spike activities corresponding to each classifying neuron in the MNIST classification network in response to the activated input image during the first and last training epochs, respectively. Figures (**c**) and (**d**) depict the count of spike activities associated with each classifying neuron in the CIFAR10 classification network in response to the activated input image during the initial and final training epochs, respectively. Upon completing the training process, the classifying neurons accurately recognize the input pattern.

During the training epochs, the image patterns are learned by the synaptic weights of the network, causing the classifying neurons to exhibit almost random responses to input stimuli in the initial training epoch. Conversely, in the last training epoch, the output layer neurons successfully classify input patterns with a high degree of accuracy. The spike activity of the output layer neurons in MNIST and CIFAR10 recognition networks in reaction to input stimuli during the initial and final epochs of training is depicted in Fig. 12.

As can be shown in Fig. 12a,c, at the start of training, by applying diverse stimulation stimuli (y-axis), irregular and random spike activity is detected in the classifying neurons of the output layer (x-axis). In contrast, when the training epochs are completed, the classifier neurons properly categorize the input stimuli, as shown in Fig. 12b,d. Sparse spike activity is an essential and notable element in the efficient and low-consumption implementation of spiking networks with on-chip learning on neuromorphic circuits. As shown in Fig. 12, we made the activity of neurons sparse during learning and testing by using a modest range of input stimulation and the suggested learning.

## Conclusion

The methodology proposed in this paper, which includes CORDIC-based hardware design, Spatial-Pow-STDP learning, and spatiotemporal SNN models, demonstrates noteworthy advancements in applications of spiking neural networks. The precise CORDIC algorithms, including Exp CORDIC, Ln CORDIC, and Pow CORDIC, exhibit high accuracy and efficiency, showcasing their applicability within neural network applications. The FPGA implementation on a Xilinx Zynq FPGA device and performance analysis substantiates the effectiveness of the proposed algorithms, revealing low average errors and elevated maximum speeds. The large-scale spatiotemporal SNN, trained using the Spatial-Pow-STDP learning mechanism, achieves superior classification accuracies on MNIST, EMNIST, and CIFAR10 datasets with reduced training epochs, highlighting its efficacy in comparison to other spiking neural networks. The results further underscore the advantages of the proposed network, such as reduced hyper-parameters, adaptability to unsupervised training, event-based operation for lower energy consumption, and suitability for implementation on low-power neuromorphic boards. The conclusion reaffirms the high accuracy and accelerated convergence speed exhibited by the proposed network and its hardware counterpart, emphasizing their potential contributions to spiking neural networks for image classification and further applications.

## Data Availability

Data would be available through corresponding author with reasonable request.
